# Supplementary material for: Automated echocardiographic detection of mitral valve prolapse and mitral regurgitation with video-based artificial intelligence algorithms
Source: Eur Heart J Digit Health. 2026 Apr 16;7(5):ztag061. doi: 10.1093/ehjdh/ztag061 (PMC13184616; doi:10.1093/ehjdh/ztag061)
Supplement: ztag061_Supplementary_Data [file ztag061_supplementary_data.docx]

**Supplementary tables**

| Supplementary table 1: Comparison of Single-view vs. Multi-view MVP models on UCSF test set | | | |
| --- | --- | --- | --- |
|  | AUC | Sensitivity | Specificity |
| A2C view model | 0.862  (0.839-0.883) | 0.694  (0.644-0.743) | 0.858  (0.849-0.867) |
| A4C view model | 0.862  (0.840-0.881) | 0.739  (0.688-0.784) | 0.801  (0.791-0.812) |
| PLAX view model | 0.879  (0.860-0.898) | 0.752  (0.705-0.799) | 0.839  (0.828-0.849) |
| Multi-view model | 0.917  (0.899-0.934) | 0.797 (0.754-0.841) | 0.893  (0.884-0.901) |
